# Fasting blood glucose in a Ghanaian adult is causally affected by malaria parasite load: a mechanistic case study using convergent cross mapping

**DOI:** 10.1186/s12936-022-04076-y

**Published:** 2022-03-18

**Authors:** Carol A. Abidha, Yaw Ampem Amoako, Richard King Nyamekye, George Bedu-Addo, Florian Grziwotz, Frank P. Mockenhaupt, Arndt Telschow, Ina Danquah

**Affiliations:** 1 Faculty of Medicine and University Hospital, Heidelberg Institute of Global Health (HIGH), Heidelberg University, Heidelberg, Germany; 2 Komfo Anokye Teaching Hospital, Kwame Nkrumah University of Science and Technology, Kumasi, Ghana; 3 Agogo Presbyterian Hospital, Agogo, Ghana; 4 Institute for Evolution and Biodiversity, Westfälische Wilhelms-Universität Münster, Münster, Germany; 5 Institute of Tropical Medicine and International Health, Charité—Universitätsmedizin Berlin, Corporate Member of Freie Universität Berlin and Humboldt-Universität Zu Berlin, Berlin, Germany; 6 Institute for Environmental Systems Research, Osnabrück University, Osnabrück, Germany; 7 Department Molecular Epidemiology, German Institute of Human Nutrition Potsdam-Rehbrücke, Nuthetal, Germany

**Keywords:** Diabetes mellitus, Malaria, Convergence cross mapping, Empirical dynamic modelling

## Abstract

**Background:**

Adults with diabetes mellitus (DM) in malaria-endemic areas might be more susceptible to *Plasmodium* infection than healthy individuals. Herein, the study was aimed at verifying the hypothesis that increased fasting blood glucose (FBG) promotes parasite growth as reflected by increased parasite density.

**Methods:**

Seven adults without DM were recruited in rural Ghana to determine the relationships between FBG and malaria parasite load. Socio-economic data were recorded in questionnaire-based interviews. Over a period of 6 weeks, FBG and *Plasmodium* sp*.* Infection were measured in peripheral blood samples photometrically and by polymerase chain reaction (PCR)-assays, respectively. Daily physical activity and weather data were documented via smartphone recording. For the complex natural systems of homeostatic glucose control and *Plasmodium* sp. life cycle, empirical dynamic modelling was applied.

**Results:**

At baseline, four men and three women (median age, 33 years; interquartile range, 30–48) showed a median FBG of 5.5 (5.1–6.0 mmol/L); one participant had an asymptomatic *Plasmodium* sp*.* infection (parasite density: 240/µL). In this participant, convergent cross mapping (CCM) for 34 consecutive days, showed that FBG was causally affected by parasite density (p < 0.02), while the reciprocal relationship was not discernible (p > 0.05). Additionally, daily ambient temperature affected parasite density (p < 0.01).

**Conclusion:**

In this study population living in a malaria-endemic area, time series analyses were successfully piloted for the relationships between FBG and *Plasmodium* sp. density. Longer observation periods and larger samples are required to confirm these findings and determine the direction of causality.

**Supplementary Information:**

The online version contains supplementary material available at 10.1186/s12936-022-04076-y.

## Background

Infectious diseases such as malaria, HIV, and tuberculosis have long been the main contributors to morbidity and mortality in sub-Saharan Africa (SSA) [1]. It is encouraging that malaria-related deaths have declined globally from 585,000 in 2010 to 405,000 in 2018 [2]. However, in 2019, malaria deaths increased to an estimated 558,000 and further to 627,000 in 2020 indicating that it still constitutes a major public health threat in SSA, with about 80% of deaths occurring among children under the age of five years [3]. In Ghana, West Africa, malaria remains highly endemic with an annual incidence of 162 per 1000 at risk [2]. Notably, it is clustered under the moderate to high transmission countries which account for 70% of the global estimated case burden and 71% of global estimated deaths [3].

At the same time, non-communicable diseases (NCDs) have gained considerable importance globally and in SSA, including an “epidemic” of diabetes mellitus (DM) [4]. Worldwide, 463 million adults are living with DM as of 2019, with 79% residing in low- and middle-income countries. The International Diabetes Federation predicts that worldwide, there will be 700 million people living with DM by 2045 [5]. Type 2 diabetes mellitus (T2DM) poses a growing health problem on the African continent [5, 6], including Ghana, where 10% of adults have T2DM [7]. The accelerating urbanization in the African region comes along with changes in dietary behaviour and physical activity, which contribute to the observed increase of T2DM [8]. In addition to this, the epidemiologic transition from infectious diseases to NCDs due to increased life expectancy and lower birth rates progresses slowly in SSA. These factors give rise to the observed double burden of infectious diseases and NCDs in the region. This double burden has been recognized at the country level [9] and among individuals [10]. Thus, interrelations of malaria and DM in SSA appear logical but have only been insufficiently investigated so far [11]. Previously, first-time data suggesting that early-life exposure to malaria may increase the risk of cardio-metabolic diseases in later life, including DM was provided [12]. In this study, the relationships between FBG and *Plasmodium* sp. density have been investigated in adults.

Previously, a 46% increased odds of *Plasmodium falciparum* infection was reported among Ghanaian adults with T2DM in urban Ghana [13]. While this observation warrants independent verification, underlying mechanisms have been proposed. First, patients with DM may be more attractive to the mosquito vector *Anopheles gambiae* due to diabetes-related alterations in olfactory signals and thus, experience increased exposure to infectious bites [14, 15]. Second, the enhanced transmission of malaria parasites to individuals with DM has been suggested, based on findings in a murine malaria model [16]. In addition to this, a recent ex vivo study has demonstrated an enhanced *P. falciparum* virulence in the blood of uninfected type 1 and type 2 diabetics[17]. Third, immune suppression among individuals with DM may compromise the clearance of infected red blood cells and thus, prolong the lifespan of the parasites [18]. Fourth, malaria parasites depend on external glucose [19], which is chronically increased in DM patients and thus, may fuel parasite growth. So far, the latter has been shown only under laboratory conditions and has not been addressed in real-life settings. At the same time, FBG homeostasis and malaria parasite density are tightly regulated [20], which hampers the investigation of diabetes-malaria-interactions in cross-sectional studies.

Therefore, the major goal of this study was to establish the causal relationships between FBG concentrations and malaria parasite density using time series analysis of a prospective observational pilot study, among adults in rural Ghana. The specific objectives were (i) to determine the time-varying associations between FBG and parasite load, and (ii) to identify causal interaction between FBG and *Plasmodium* parasite density. Here, the term causality is employed in relation to statistics and time series. A modern method for testing causal relationships was used [21], which, in contrast to the classical approaches of economics [22], is tailored to research questions in life sciences.

## Methods

### Study design and procedures

From September to October 2019, a prospective observational pilot study with seven adults who resided in Agogo, Ashanti Akim North District, Ashanti Region, Ghana, was conducted. Subsistence farming, trading, and mining are the main income sources in this region [23]. Eligible individuals were recruited in the vicinity of Agogo Presbyterian Hospital and participated in daily follow-up visits over 6 weeks, except on Sundays. Inclusion criteria comprised adult age, permanent residence in Agogo, and written informed consent. Exclusion criteria were plans to travel within the following 6 weeks, known diagnosis of DM, taking glucose-lowering medication, and known pregnancy. Following detailed information on the background and procedures of the study, appointments at the hospital were scheduled for the next morning. The participants were instructed on overnight fasting and avoiding physical activity for at least 8 h before visiting the hospital. The individual examination comprised the daily collection of fasting peripheral blood samples for glucose measurement and malaria detection every morning, measurement of axillary temperature, and documentation of daily weather conditions and physical activity. Baseline interviews were conducted to assess the participants’ socioeconomic backgrounds.

The study protocol adhered to the principles laid down in the Declaration of Helsinki and was reviewed and approved by the Committee on Human Research, Publication and Ethics of the School of Medical Sciences/Komfo Anokye Teaching Hospital, Kwame Nkrumah University of Science and Technology, Kumasi, Ghana (CHRPE/AP/507/18). All participants gave written informed consent.

### Glucose measurement and malaria detection

FBG was measured in finger-prick fasting blood samples applying a portable HemoCue Glucose 201^+^ RT device (HemoCue, Germany), providing plasma equivalents of FBG in mmol/L. Malaria parasites were microscopically identified on Giemsa-stained (4%, 30 min, pH 7.2) thick and thin blood films. Parasite density was quantified by expert microscopists who examined microscopy fields of the thick film corresponding to 500 white blood cells (WBCs); the average WBC count was set as 8000/µL. Thin films served for species differentiation. In addition, venous whole blood samples were collected on filter paper (Whatman cellulose chromatography paper, 3 mm). DNA was extracted using the QIAmp DNA Blood Mini kit (QIAGEN, Germany https://www.qiagen.com). *Plasmodium* species and sub-microscopic infections were then identified by semi-nested multiplex PCR assays [24]. For further quantification, also of submicroscopic *Plasmodium* infections, crossing point (Cp) values of quantitative real-time PCR analyses were used, using commercially available primers and probes for *Plasmodium falciparum*, *Plasmodium vivax*, *Plasmodium ovale*, and *Plasmodium malariae *(TIB MolBiol, https://www.tib-molbiol.com) on a Roche LightCycler 480 device (https://lifescience.roche.com). In that, a high Cp value corresponds to a low parasite load, and vice versa. *Plasmodium* infection was defined by the more sensitive PCR assay in the absence of current fever (axillary temperature ≥ 37.5 °C) and was not treated during the present study. Clinical malaria was defined as microscopically visible parasites plus current fever and was treated according to Ghana Health Service guidelines.

### Assessment of covariables

Demographic and socioeconomic data were documented in face-to-face interviews by trained study personnel and comprised age (years), gender (m/f), residence (Agogo or else), known DM status (yes/no/unknown), known malaria status (yes/no/unknown), level of formal education (none/primary/secondary/tertiary/other), current occupation (subsistence farming/trading/business/public servant/unemployed), and the number of household’s physical assets (list of 11 items). Further, meteorological data and physical activity levels were assessed as factors potentially influencing both, FBG [25] and parasite density [26, 27]. Each participant was supplied with a standard smartphone and pre-installed applications for the assessment of these covariables. Daily air humidity (%) and ambient temperature (°C) (accuweather.com), as well as the duration of daily physical activity (min/d) and the step count (steps/d) (Google Fit) were documented.

In addition, body weight to the nearest 100 g and height to the nearest cm were assessed in duplicates, using the Person Scale DT602 (Camry, Hong Kong, China) and the statometer SECA 213 (Hamburg, Germany), respectively. Body mass index (BMI) was calculated as weight (kg) over squared height (m) and is expressed in kg/m^2^.

### Data analysis

The baseline characteristics of the study population are presented as median and range for continuous variables and as absolute numbers for categorical data.

For establishing causal links between FBG and malaria parasite load, linear statistical methods such as correlation analysis were inappropriate. In fact, the metabolic processes regulating blood glucose and parasite density over time are inherently non-linear [28]. Also, linear analyses had the risk of detecting spurious correlations, which might have led to wrong or misleading conclusions [21, 29, 30]. To overcome the problematic setting of cross-sectional assessments for the investigation of time-varying fluctuations in glucose metabolism and parasite growth, the framework of empirical dynamical modelling (EDM) that acknowledges non-linear dynamics was applied [31]. Here, Convergence Cross Mapping (CCM) was used for the identification of causal coupling between FBG and malaria parasite count [21]. More detail about this method is presented in the Additional file 1. In brief, CCM operates on the theory of dynamical systems. This theory acknowledges complex biological interactions in time and space that can be mathematically detected [32]. Following this idea, investigations were carried to find out if FBG and parasite load as two-time series variables were causally coupled (originated from the same dynamical system), by measuring the extent to which the time series of the causal variable has left an imprint in the time series of the affected variable [21]. FBG concentration was defined as the causal variable, and *P. falciparum* density served as the affected variable (here: Cp as a proxy measure).

For significance testing, 100 surrogate time series that were comparable to the measured data but fulfilled the null hypothesis of no relationship between the two, time series variables were generated [33], following the method by Ebisuzaki [34]. The causal couplings of the empirical time series were defined as significant if the CCM result of the original data outperformed 95% of the CCM results of the surrogate data (p < 0.05) [29, 35]. Furthermore, the embedding dimension (*E*) for each potential causal combination (from FBG, weather parameters, and body temperature to parasite density) in CCM analysis was determined following the procedure of Deyle and colleagues [30]. This corresponds to a lag nonlinear model, where cross-mapping *ρ* lagged 1-time step for the largest possible number of points in the surrogate time series. Further, all-time series were normalized to zero mean and unit variance. Due to the paucity of observation points, a leave-one-out cross-validation was performed [36]. Two missing values for parasite density were imputed by linear interpolation. Time series analysis was performed using the rEDM package (version 0.7.3) of the programming language *R* [37].

## Results

### Study population

Table 1 presents the baseline characteristics of the study population. Among the seven participants, there were four men and three women with an age range between 30 and 48 years. Six participants reported belonging to the Akan tribe. Five individuals permanently resided in Agogo, and six participants had secondary formal education. All participants worked as sellers or other service personnel in the vicinity of the hospital. The median of household assets was 6 out of 11. The BMI ranged between 20.7 and 39.1 kg/m^2^, and four individuals had a BMI ≥ 25.0 kg/m^2^ One woman presented with a history of gestational diabetes. The baseline axillary body temperature was in the normal range for all participants (median: 36.3 °C; range: 35.3–36.7 °C). Also, the baseline FBG was in the normal range (median: 5.5 mmol/L; range: 5.1–6.0 mmol/L).

**Table 1:** Baseline characteristics of 7 study participants under 6-weeks glucose- and *Plasmodium*-monitoring

Characteristics	Median (range)/n
N	7
Age (years)	33 (30–48)
Males:females	4:3
Agogo residence	5
Akan ethnicity	6
Secondary education	6
Occupation	
Street vendor	2
Cleaner	2
Security service	2
Public servant	1
No. of 11 assets	6 (2–7)
Body Mass Index (kg/m^2^)	27.8 (20.7–39.1)
BMI ≥ 25.0 kg/m^2^	4
Baseline fasting plasma glucose (mmol/L)	5.5 (5.1–6.0)
Baseline axillary temperature (°C)	36.3 (35.3–36.7)
Baseline *P. falciparum* infection	1
Baseline malaria parasite count of infected individuals (GMPD/µL)	240
Baseline physical activity (min/d)	61.8 (16.2–114.6)
Baseline step count (steps/d)	5728 (1664–11,072)

**Fig. 1:**
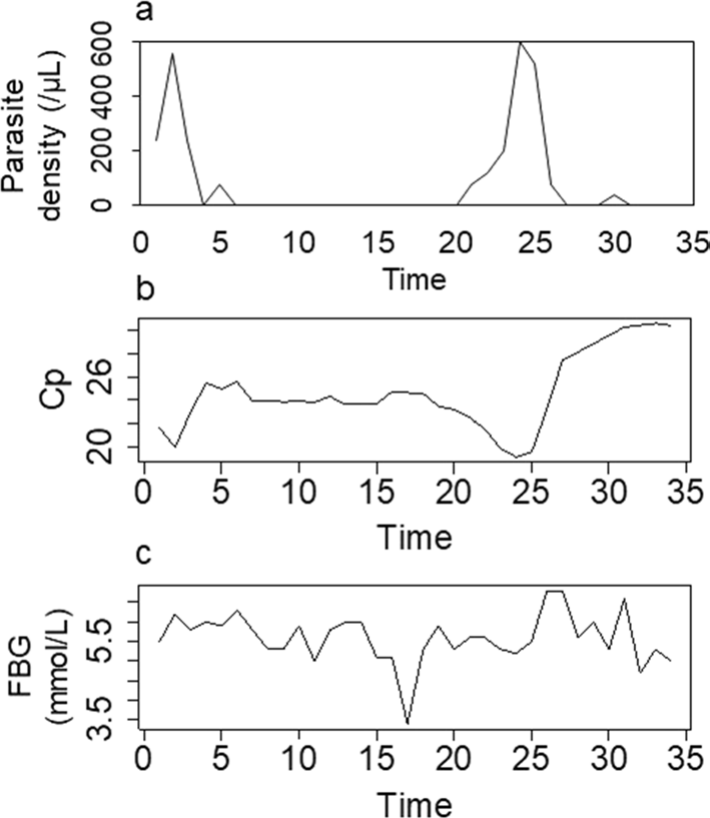
Empirical time series of participant #2. Shown are **a** *Plasmodium falciparum* density, **b** Crossing point (Cp) as a negative correlate of parasite density by PCR, and **c** fasting blood glucose (FBG), as a function of time

Only one individual had microscopically visible *P. falciparum* infection at low parasite density (baseline: 240 parasites/µL). This infection was asymptomatic throughout the study period. As shown in Fig. 1a, this person had a microscopically visible infection on 10 non-consecutive days and presented two peaks in microscopically detected parasite density on day 2 (560 parasites/µL) and day 24 (600 parasites/µL). Corresponding Cp values as a measure of (submicroscopic) parasite load showed similar valley time points and constant parasite load throughout the study (Fig. 1b). For this participant, baseline FBG was normal (5.5 mmol/L) and varied thereafter between 3.0 and 6.8 mmol/L (Fig. 1c). None of the remaining participants was subsequently infected during the study period. The variation in FBG was similar among all individuals (Additional file 1: Table S1).

### Relationships of fasting blood glucose with malaria parasite density

**Fig. 2:**
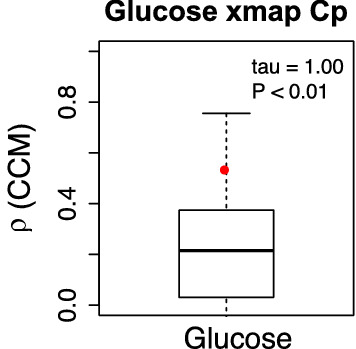
Convergence cross-mapping (CCM) detects a causal effect of Cp (= measure of *Plasmodium falciparum* density) on fasting blood glucose concentration in patient #2. Shown are the CCM results $\rho$ of the empirical time series (red dot) as well as the results of the surrogate time series (box plot). The empirical result $\rho$=0.538 outperforms over 95% of the surrogates. A significant Kendall’s $\tau$ quantifies the convergence of $\rho$ for increasing library size

For the one participant with asymptomatic malaria infection, Cp values remained stable when FBG ranged between 4.5 and 5.5 mmol/L. The time series of FBG and parasite density (Fig. 1a, b) indicated that that FBG fluctuated around the equilibrium. This was also discernible for the relationship between FBG and Cp values (Fig. 1a, c). The results of the CCM analysis indicated that Cp causally affected FBG concentration (p < 0.01) but not vice versa (Fig. 2). The surrogate data for no relationship between Cp and FBG were outperformed by 95% of the empirical time series for these two variables.

### Relationships with covariables

**Fig. 3:**
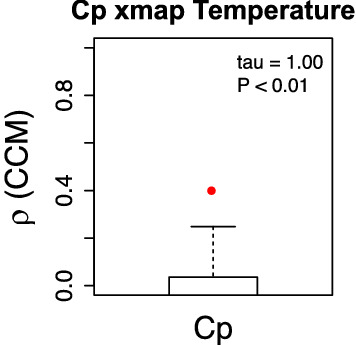
Convergence cross-mapping (CCM) detects a causal effect of temperature on Cp (measure of *Plasmodium falciparum* density)

To test the robustness of the CCM analysis in this dynamical system of FBG homeostasis and regulation of parasite density, additional relationships with relevant covariables were examined. Regarding causal links with Cp values, weather parameters and body temperature were assessed for the participant with asymptomatic malaria infection. The mean daily ambient temperature was 25.2 °C (SD = 1.9 °C) with the maximum recorded being 31 °C and the minimum 23 °C. The results of the CCM analysis for air temperature and Cp values in this participant are shown in Fig. 3. Cp lagged daily ambient temperature (p < 0.01). The mean relative humidity was 84.0% (SD = 9.1%) ranging between 97 and 61%. There was no relationship between humidity and Cp (p > 0.05). Also, in this asymptomatic participant, a causal relationship between body temperature and Cp was not detected (p > 0.05).

Regarding causal relationships with FBG, the duration of physical activity (min/day) and step count (steps/day) among all study participants were analyzed. As shown in Table 2, various lag times were tested (0–5 days). Among four participants, the empirical CCM results outperformed the surrogate time series for physical activity and FBG, indicating that both, duration of physical activity and step count, affected FBG (p < 0.05).

**Table 2:** Convergence cross-mapping detects causal effects of physical activity (min/day) and step count (steps/day) on fasting blood glucose (FBG) concentration

Patient number	Affected variable	Effecting variable	Time lag (day)	Kendall’s $\tau$	*p*_*τ*_
1	FBG	Activity in min	0	0.929	< 0.0001
1	FBG	Activity in min	3	1	< 0.0001
1	FBG	Step count	0	0.937	< 0.0001
1	FBG	Step count	3	1	< 0.0001
2	FBG	Activity in min	2	0.974	< 0.0001
2	FBG	Step count	2	0.974	< 0.0001
3	FBG	Step count	1	0.918	< 0.0001
6	FBG	Step count	5	1	< 0.0001
7	FBG	Activity in min	5	1	< 0.0001
7	FBG	Step count	5	1	< 0.0001

### Discussion

In this prospective observational study in rural Ghana, the causal relationships between the time series of FBG and measures of malaria parasite load was analysed. During the study, only one out of seven participants had an asymptomatic malaria infection at low parasite density. In this participant, CCM results showed that Cp values (as a measure of submicroscopic parasite density) affected FBG concentrations. But the effect of FBG on Cp could not be detected. In addition, temperatures affected parasite density, and physical activity affected FBG.

### Relationships of fasting blood glucose with malaria parasite load

The results agree with the fact that *Plasmodium* sp. lacks the ability to store energy in the form of polysaccharides such as glycogen. Therefore, the parasite relies on an exogeneous glucose supply [38–42]. With enough glucose supply, parasite growth and proliferation are enhanced. Vice versa, when glucose supply is interrupted or drops below 5.5 mmol/L, parasite growth and proliferation are greatly impaired [43], giving further weight to the finding that Cp values remained stable when FBG ranged between 4.5 and 5.5 mmol/L. Interestingly, to ease the availability of glucose, infected erythrocytes exhibit a substantial increase in erythrocyte membrane permeability to low molecular weight sugar. In fact, these erythrocytes utilize up to two orders of magnitude more glucose than their non-infected counterparts [40, 44, 45], leading to hypoglycaemia—a common symptom of clinical malaria [20]. In addition, it is well-established that inflammatory processes during asymptomatic malaria infection induce hypoglycaemia [46].

Even though CCM did not detect the reciprocal relationship between FBG and Cp, this observation could be explained by the fact that it was only possible to detect and follow-up malaria parasite load in one participant, thereby increasing the chances for type II error. Nonetheless, increased glucose production and higher FBG in patients with uncomplicated falciparum malaria, possibly driven by increased hepatic gluconeogenesis in the host, have been previously reported [47–49]. In addition to this, studies have shown that glucose production is even more pronounced in adults with severe malaria [50]. Clearly, the results of the present study need to be verified in a larger sample and with a longer observation period. Still, it remains plausible that individuals with regularly increased blood glucose concentration might experience enhanced malaria parasite growth as compared to subjects with healthy glucose metabolism.

### Interrelations with weather parameters and physical activity

The relationships between weather parameters and parasite load have not been sufficiently explored. However, in SSA, a statistically significant variation of mean parasite density (p value < 0.01) has been previously reported to be influenced by different seasons [51]. In the present study, daily ambient temperatures were associated with Cp. Further CCM confirmed the causality of this relationship, pointing towards an important role of temperature in parasite growth. Therefore, this weather parameter could modulate the relationship between FBG and Cp. In the human host, heat induces vasodilation, which leads to enhanced glucose uptake into the peripheral tissues and thus, reduced FBG [52]. In the *Anopheles* vector, ambient temperature modifies the interaction between gametocyte density and infectivity to the mosquito vector [26].

Also, the observed relationship between physical activity and FBG accords with existing scientific evidence. Physical activity has a glucose-lowering effect [53] wherefore it is recommended for the prevention of T2DM and as one of the first-line T2DM treatments [54]. Physical exercise induces glucose uptake into skeletal muscles [55], and supports the stabilization of plasma blood glucose in the postprandial response, thereby limiting hyperglycemic peaks [56]. The absence of a significant coupling between physical activity and FBG by CCM analysis in some participants might stem from the limited number of data points and potential process/observation noise.

Pending verification of the hypothesis, individuals with chronically increased blood glucose need to be advised to strongly adhere to malaria protective measures. This may have implications for patients with poor blood glucose control living in SSA. In fact, only 37% of adults with T2DM in rural Ghana receive glucose-lowering medication, and of these, only 63% have good glucose control [57]. In addition, children with more severe type 1 DM may be particularly affected, because poor glucose control and lack of semi-immunity against malaria parasites may support the proliferation of *Plasmodium* sp. Lastly, diabetes has been indicated to be a key risk factor for severe malaria infection [58]. Travelers with DM should, therefore, be encouraged to adhere to their glucose-lowering medication and malaria prophylaxis.

### Strengths and limitations

This is the first study, in which time series analysis has been applied, to determine the causal relatedness of FBG to *Plasmodium* sp. density. However, the results of the study need to be interpreted with caution. Even though the number of participants was fair enough for time series analysis, only one individual developed *Plasmodium* sp. infection, thereby leading to type II error and thus limiting the ability to verify the hypothesis that higher FBG leads to higher parasite load. No examinations were conducted on Sundays, which interrupted the time series assessments. Yet, for the participant with asymptomatic infection, any issues arising from missing data were overcome by leave-one-out cross validation and linear interpolation. Still, independent studies with longer observation periods and larger sample sizes are warranted for a better in-depth explanation of this causal relationship and to verify the direction of causality.

## Conclusion

In conclusion, CCM has been applied to investigate whether FBG among Ghanaian adults is causally linked with malaria parasite density. Since metabolic processes regulating blood glucose and malaria parasite growth are inherently non-linear, the EDM framework for non-linear dynamics in time series data was utilised. In this healthy study population without DM, the common observation of malaria-related hypoglycemia has been verified. Yet, the confirmation of the original hypothesis is pending until longer and larger time series analysis can provide causal links between chronically increased FBG and malaria parasite growth.

## Supplementary Information

**Additional file 1: Table S1. **FBG parameters for all patients. Note that only patient 2 was infected with *Plasmodium falciparum. *Comparing variability (quantified by the standard deviation, SD) of infected and uninfected patients using Shapiro-Wilk-Test with Levene’s Test revealed that variability does not differ significantly (P > 0.05).

## Data Availability

The datasets generated and/or analysed during the current study are available from the corresponding author on reasonable request.
