# Supplementary material for: Fasting blood glucose in a Ghanaian adult is causally affected by malaria parasite load: a mechanistic case study using convergent cross mapping
Source: Malar J. 2022 Mar 18;21:93. doi: 10.1186/s12936-022-04076-y (PMC8932323; doi:10.1186/s12936-022-04076-y)
Supplement: Supplementary file 1 — Additional file 1: Table S1. FBG parameters for all patients. Note that only patient 2 was infected with Plasmodium falciparum. Comparing variability (quantified by the standard deviation, SD) of infected and uninfected patients using Shapiro-Wilk-Test with Levene’s Test revealed that variability does not differ significantly (P > 0.05). [file 12936_2022_4076_MOESM1_ESM.docx]

**Supplementary material – Methods**

***Convergence cross-mapping***

According to the theory of dynamical systems, time series are projections of the motion on a multidimensional (given by the number of interacting variables) manifold following a specific set of rules onto a coordinate axis. Conversely, observed time series can be projected back into a multidimensional state space to recover the dynamics, which is known as attractor reconstruction [1]. As a consequence, even though the set of rules governing a dynamical system is not known, the system can be constructed by plotting all-time series as the axes in a state space and view how the state evolves through time.

In empirical sciences such as biology and medicine, however, one is neither able to measure the multidimensional manifold given by the underlying rules nor know all critical variables of the system. Most of the time, only one or two time series of the studied system is observed. Therefore, it is possible to lack observation data necessary to reconstruct the dynamics. Surprisingly, the original process can be reconstructed from time series observations of a single variable by embedding time-delayed values of the scalar measurements, since the information of all unknown variables are encoded in every single observed time series [2]. Consequently, even if the observed time series variable depends on variables that were not measured, a shadow version of the manifold that accounts for these missing variables can still be reconstructed.

The reconstructed shadow manifold as well as the original manifold are one-to-one mappings. As a result of this, they share the same mathematical features such as topology or lyapunov exponents [3]. For instance, consider two time series, X and Y, originating from the same dynamical system. If the reconstructed manifold of time series X is invariant of the original manifold and at the same time, the reconstructed manifold of time series Y is invariant of the original manifold, both reconstructions have to be invariant to each other. Following this idea, the investigation on whether two time series variables are causal coupled (originate from the same dynamical system) is conducted by measuring the extent to which the causal variable has left an imprint, in the time series of the affected time series, using convergence cross mapping (CCM) [4]. In this study, causal effects between Cp as a measure of *P. falciparum* density (Cp), FBG concentration as well as environmental climate variables were tested.

***Convergence and significance***

To test time series findings for significance, a number of surrogate data sets which are comparable to the measured data in certain respects, but which are also consistent with the null hypothesis under test, were used [3]. Here, 100 surrogate time series were generated, following [5], by randomly reshuffling phases, while preserving the mean, variation and power spectrum of the original time series. The causal couplings of the empirical time series were deemed significant if the CCM result of the original data outperformed 95% of the CCM results of the surrogate data (P < 0.05) [6,7].

Furthermore, the embedding dimension (*E*) for each causal link (e.g. from *Y* to *X*) in CCM analysis was determined by testing values of *E* from 1 to 8 dimensions following the procedure of Deyle [8]. Thus, dimension *E* was chosen that optimizes cross-mapping *ρ* lagged 1-time step for the largest possible number of points in the library. Further, all-time series were normalized to zero mean and unit variance so that the Euclidian distances on the reconstructed manifolds are scale-free [8]. Due to the paucity of observation points, leave-one-out cross-validation was performed [9]. Two missing values for parasite density were imputed by linear interpolation. Time series analysis was performed using the rEDM package (version 0.7.3) of the programming language *R* [10].

**Supplementary material references**

1. Packard NH, Crutchfield JP, Farmer JD, Shaw RS. Geometry from a time series. Phys Rev Lett. 1980;45:712–6.

2. Takens F. Dynamical systems and turbulence. In: Rand DL, Young LS, Eds. - Detecting strange attractors in turbulence. Springer Link. 1981;898:366-81.

3. Kantz H, Schreiber T. Nonlinear Time Series Analysis. 2nd Edn. Cambridge University Press, 2003.

4. Sugihara G, May R, Ye H, Hsieh CH, Deyle E, Fogarty M, et al. Detecting causality in complex ecosystems. Science. 2012;338:496-500.

5. Ebisuzaki W. A method to estimate the statistical significance of a correlation when the data are serially correlated. J Climate. 1997;10:2147-53.

6. Deyle ER, Fogarty M, Hsieh CH, Kaufman L, MacCall AD, Munch SB, et al. Predicting climate effects on Pacific sardine. Proc Natl Acad Sci USA. 2013;110:6430-5.

7. Van Nes EH, Scheffer M, Brovkin V, Lenton TM, Ye H, Deyle E, et al. Causal feedbacks in climate change. Nat Clim Chang. 2015;5:445-8.

8. Deyle ER, Maher MC, Hernandez RD, Basu S, Sugihara G. Global environmental drivers of influenza. Proc Natl Acad Sci USA. 2016;113:13081-6.

9. Glaser SM, Fogarty MJ, Liu H, Altman I, Hsieh CH, Kaufman L, et al. Complex dynamics may limit prediction in marine fisheries. Fish Fisheries. 2014;15:616-33.

10. Ye H, Clark A, Deyle E, Sugihara G. rEDM: an R package for empirical dynamic modeling and convergent cross-mapping. R-ProjectOrg. 2020.
